# Reproductive potential does not cause loss of heat shock response performance in honey bees

**DOI:** 10.1038/s41598-020-74456-4

**Published:** 2020-11-12

**Authors:** S. R. Shih, E. M. Huntsman, M. E. Flores, J. W. Snow

**Affiliations:** Biology Department, Barnard College, New York, NY 10027 USA

**Keywords:** Physiology, Cell biology, Cell signalling, Protein folding

## Abstract

In other species characterized to date, aging, as a function of reproductive potential, results in the breakdown of proteaostasis and a decreased capacity to mount responses by the heat shock response (HSR) and other proteostatic network pathways. Our understanding of the maintenance of stress pathways, such as the HSR, in honey bees, and in the reproductive queen in particular, is incomplete. Based on the findings in other species showing an inverse relationship between reproductive potential and HSR function, one might predict that that HSR function would be lost in the reproductive queens. However, as queens possess an atypical uncoupling of the reproduction-maintenance trade-off typically found in solitary organisms, HSR maintenance might also be expected. Here we demonstrate that reproductive potential does not cause loss of HSR performance in honey bees as queens induce target gene expression to levels comparable to those induced in attendant worker bees. Maintenance of HSR function with advent of reproductive potential is unique among invertebrates studied to date and provides a potential model for examining the molecular mechanisms regulating the uncoupling of the reproduction-maintenance trade-off in queen bees, with important consequences for understanding how stresses impact different types of individuals in honey bee colonies.

## Introduction

Aging is associated with the progressive breakdown of the proteome, resulting in cellular dysfunction and physiological decline affecting multiple systems^1^. At the cellular level, inadequate capacity of the protein synthesis machinery, quality control and folding pathways, degradation machinery, and stress responses of the proteostatic network result in the loss of proteostasis^2–4^. Proteostatic network decline with aging in invertebrates can be divided into two stages. The first stage of aging appears to be set in motion by programmed life events that occur with the onset of reproduction. The second phase involves a slowly building loss of function that is associated with chronological age and the process of senescence^2–4^**.** Protein aggregation in diverse tissues increases with age in *Caenorhabditis elegans*^5–7^ and *Drosophila melanogaster*^8^. Concomitantly, researcher have observed reduced protein synthesis^9^ and degradation through the Ubiquitin Proteasome System (UPS)^10,11^. Studies have also found age-dependent reduction in the pathways of the proteostasic network, including the Hat Shock Response (HSR), responding to proteotoxic stress in the cytoplasm, in *C. elegans*^2^ and *D. melanogaster*^12^. This age-related loss of HSR potential after reaching adult form observed in *C. elegans* appears to be a highly regulated event that coincides with the onset of reproductive potential^2^ and can be delayed by removal of germ cells^13^.

Studies in model invertebrates have played a key role in elucidating the molecular, cellular, and organismal determinants of aging described above through the Ubiquitin Proteasome System (UPS)^14,15^. However, aging in social insects, such as the honey bee, differs considerably from these solitary model organisms, which has made it an attractive model for understanding aging^16^. First, due to the division of labor, the sterile worker bees, which make up the vast majority of colony members, are non-reproductive except in rare cases and each colony typically has a single reproductive female queen. Remarkably, the queen is the most long-lived individual by an order of magnitude. Second, in addition to chronological aging, the non-reproductive worker caste of honey bees exhibit a phenomenon known as age polyethism, or the age-related division of labor for non-reproductive tasks (manifested through both behavioral and physiological changes)^17–19^. The plasticity of the transitions in age polyethism are of particular note. While the HSR, responding to proteotoxic stress in the cytoplasm, has been well characterized in the invertebrate models, *D. melanogaster* and *C. elegans*^20,21^, our understanding of this pathway in honey bees is incomplete. Furthermore, there is little information on how chronological aging, age polyethism, or reproductive potential affect such stress response pathways as the HSR in this species.

In particular, our knowledge on the function of these stress pathways, including the HSR, in the reproductive queen is incomplete. Queens might be expected to maintain HSR function despite being reproductive. They are the most long-lived individual in the colony and this longevity appears to be the result of uncoupling of the reproduction-maintenance trade-off typically found in solitary organisms. However, based on the findings in other species showing an inverse relationship between reproductive potential and HSR function^2–4^, one might predict that that HSR function and reproduction are decoupled in honey bees through the caste system, such that sterile workers maintain HSR function, while reproductive queens lose it. An additional rationale for queen loss of HSR is the colony’s remarkable thermostasis. Colony temperature is carefully maintained between 32° and 35 °C during normal conditions in colonies in temperate regions through the actions of worker bees^22–26^. This narrow temperature range is important for brood development and normal colony function and may allow queens to give up HSR function in the context of this relatively thermal stress-free environment.

By contrast, honey bee workers are exposed to routine extreme heat stresses brought on by normal honey bee foraging activity^27^, aggressive activity^28^, and maintenance of colony thermostasis^25^. The temperature of individual worker bees can increase significantly above steady-state to levels that are dangerous to other organisms. For example, the temperatures of individual forager bees can reach up to 49 °C in flight^27^ and bees engaged in endothermic heat production reach high temperatures as well. Individual honey bees workers appear to have a high capacity to endure thermal stress^29–33^. One reason for this high-level resilience is likely their robust HSR^34^, which contributes to thermotolerance at the cellular level in most species. In a recent study, we showed that the core molecular components, target genes, and function of the HSR are conserved in honey bees workers^35^.

Here, we explored whether reproductive honey bee queens have a reduced capacity to induce the protective HSR in response to heat stress in comparison to sterile workers. Our results demonstrate that reproductive potential does not cause loss of HSR performance in honey bees. Specifically, honey bee queens induce HSR target gene expression to levels comparable to those induced in attendant worker bees. Maintenance of HSR function with advent of reproductive potential observed here in honey bees is unique among invertebrates studied to date. Thus, this system provides a potential model for examining the molecular mechanisms regulating the uncoupling of the reproduction-maintenance trade-off in queen bees and may also have important consequences for understanding how stresses impact different types of individuals in honey bee colonies.

## Results

### Queen tissues possess a robust HSR

**Figure 1:**
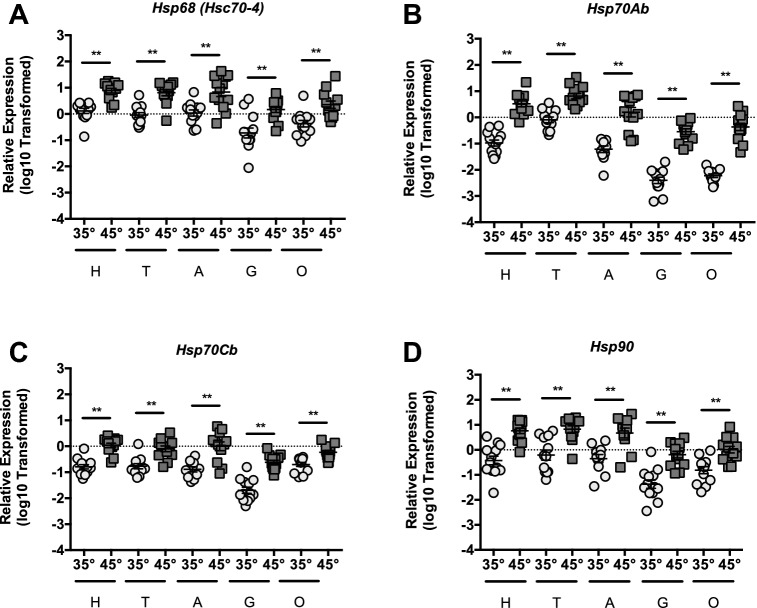
HSR target genes are induced during heat-shock in honey bee queens. Transcript levels of HSR target genes *Hsc70-4* (**A**), *Hsp70Ab* (**B**), *Hsp70Cb* (**C**), and *Hsp90* (**D**), relative to *β-actin* in head tissue (H, predominantly brain and sensory organ tissue) thorax tissue (T, predominantly flight muscle), abdominal wall tissue (A, predominantly fatbody), midgut (G), and ovarian tissue (O) from queen bees maintained for 4 h in cages at either 35° or 45 °C. Symbols represent expression values of the genes of interest calculated using the ΔΔC_T_ method for individual bees and Log10 Transformed. Individual values and mean ± SEM are also shown. Statistical significance was assessed using unpaired t-tests with Welch’s correction as values fit normal distributions and is noted as *p < 0.05, and **p < 0.01.

We examined heat-shock dependent induction of putative heat-shock genes in head tissue (predominantly brain and sensory organ tissue), thorax tissue (predominantly flight muscle), abdominal wall tissue (predominantly fat body), midgut, and ovarian tissue from queen bees maintained at 35° and 45 °C for 4 h. Relative to *β-actin*, we observed robust induction of the homologs of the core HSF target genes, *Hsc70-4* (Fig. 1A), *Hsp70Ab* (Fig. 1B), *Hsp70Cb* (Fig. 1C), and *Hsp90* (Fig. 1D), in all tissues examined. In addition, although we observed a robust induction of these heat-shock target genes in all five tissues, the magnitude of induction differed between tissues.

We also measured the expression of these same genes for the same tissues from attendant worker bees maintained with these queens at 35° and 45 °C for 4 h. As observed before^35^, we found strong induction of the HSR target genes, *Hsc70-4* (Suppl Fig. 1A), *Hsp70Ab* (Suppl Fig. 1B), *Hsp70Cb* (Suppl Fig. 1C), and *Hsp90* (Suppl Fig. 1D), in all tissues examined relative to *β-actin*.

#### Queen and attendant HSR are similar in magnitude with some tissue- and gene-specific differences

**Figure 2:**
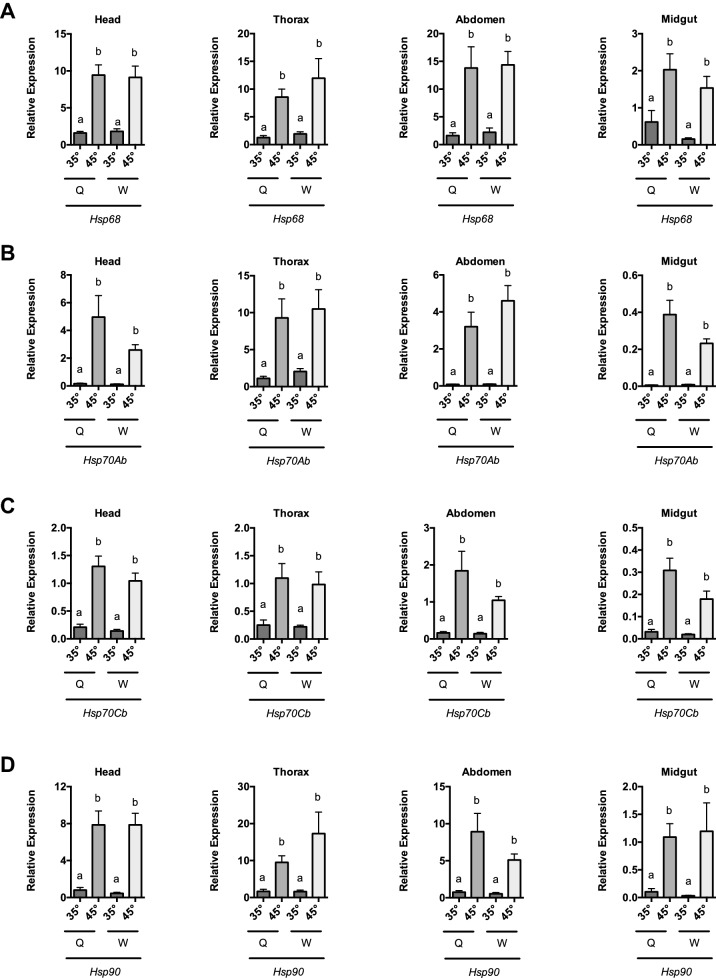
Comparable HSR gene induction in honey bee queens and attendant worker bees after heat shock. Transcript levels of HSR target genes *Hsc70-4* (**A**), *Hsp70Ab* (**B**), *Hsp70Cb* (**C**), and *Hsp90* (**D**), relative to *β-actin* from queen bees and attendant worker bees maintained for 4 h in cages at either 35° or 45 °C for head tissue (predominantly brain and sensory organ tissue), thorax tissue ( predominantly flight muscle), abdominal wall tissue (predominantly fatbody), and midgut. Expression values of the genes of interest were calculated using the ΔΔC_T_ method for individual bees and mean ± SEM is shown. Statistical significance was assessed using ANOVA on log10 Transformed data and is noted as a ≠ b, *p* < 0.01. For detailed ANOVA results, see in Statistical Data Table, Suppl Table 2.

We then compared the baseline and induced levels of the HSR target genes from queens and attendants in Fig. 1. We found that expression levels of *Hsc70-4, Hsp70Ab, Hsp70Cb,* and *Hsp90* were not different for queens and sterile attendant workers in the uninduced (35°) and induced (45°) states in head tissue, thorax tissue, abdominal wall tissue, and midgut (Fig. 2A,B,C,D) (ANOVA results in Statistical Data Table, Suppl Table 2). Analysis of the magnitude of gene induction revealed equal levels of gene induction between queens and workers for 13 of 16 comparisons made in the study (data not shown). For the other 3, we found modest changes in induction in a tissue-specific manner that did not consistently favor workers or queens. *Hsp68* gene induction was fourfold higher in the worker midgut. By contrast, the *Hsp70Ab* gene induction was twofold higher in the queen midgut. In addition, we found a twofold increase in induction in *Hsp90* gene expression in the worker head (data not shown).

## Discussion

In honey bees, we find that reproductive potential does not cause loss of HSR performance. Honey bee queens have baseline levels and induced levels of gene expression of a number of HSR target genes comparable to those induced in attendant worker bees. HSR function is maintained in both castes despite the existence of many well-defined differences in the physiology and behavior of queens and workers that are the result of different developmental programs and gene expression patterns. It is important to note that we looked at only a few of the known HSR targets as proxy for HSR function and that the number of total induced genes or the magnitude of increased expression in other genes could be different between queens and workers. However, other studies looking at aging have also focused on a few genes (for example, see^5^ where the authors examined two Hsp70 genes (*C12C8.1, F44E5.4*) and two sHSP genes (*hsp16.2*, *hsp16.11*). Analysis of the magnitude of gene induction revealed equal levels of gene induction between queens and workers for 13 of 16 comparisons made in the study. For the other 3, we found modest changes in induction in a tissue-specific manner, consistent with tissue specific nature of stress responses observed in other organisms^36^. While these changes may be biologically relevant and worthy of further study, we believe the induction data again supports our model that there is no comprehensive loss of HSR function in the reproductive queens.

Honey bees display remarkable caste-specific differences in longevity, with reproductive queens living 1–2 years, while sterile workers live 2–6 weeks in the summer and ~ 20 weeks in the winter^16,37,38^. Likely underlying this longevity, honey bee queens appear to display uncoupling of the reproduction-maintenance trade-off typically found in solitary organisms. Multiple studies show that they exhibit fewer features of senescence commonly associated with aging when compared to sterile workers. Evidence of molecular damage, such as ubiquitination of proteins are not increased in honey bee queens and the ubiquitin proteasome system appears intact despite aging^39^. Some evidence shows that queens are more resistant to oxidative stress than workers^40^, despite losing expression of antioxidant genes with age^41^, perhaps due to higher peroxidation‐resistant membranes^42^. In flight muscle basal expression of select HSR genes did not decrease with age in queens^43^. Cellular regeneration, as assessed by replicative activity of intestinal stem cells, also did not decrease with age in honey bee queens^44^. Finally, queen bees also maintain humoral immunity during aging, although they do lose cellular immunity like workers^45^.

A number of pathways appear to affect aging and lifespan in other model organisms^46^, all of which are intimately involved in metabolism at the cellular and organismal levels^47^. These include nutrient sensing pathways such as the Insulin/insulin-like growth factor-1 signaling (IIS) pathway and the Target of Rapamycin (TOR) pathway as well as mitochondrial biogenesis and function. In *C. elegans*, both IIS and TOR pathways^48^ and mitochondrial function^49^ impact the function of Heat Shock Factor (HSF). All three of these pathways have been shown to be involved in queen development or longevity^40,50–56^. However, the dominant theory explaining this atypical association between reproductive fecundity and longevity is the altered wiring of the IIS-JH-Vg circuit^57^. In the solitary model organisms, IIS activates JH production which promotes Vg transcription in response to a high nutritional state, leading to pro-reproductive and pro-aging effects at the expense of maintenance (such as the preservation of stress responses) and survival effects. The physiological state of the organism switches to more pro-maintenance and pro-survival effects at the expense of reproduction in response to certain environmental stimuli, such as nutrient deprivation^58^. In honey bees, a number of changes to the relationships of components of this circuit are thought to occur with the main effects being (1) a decoupling of Vg production from nutritional status such that it is constitutively produced in adult queens and (2) that the physiological effects of Vg is to simultaneously promote pro-reproductive and pro-maintenance effects in honey bee queens. Additional studies could be performed to explore whether this rewiring contributes to the maintenance of HSR in honey bee queens.

Proteostatic network decline with aging in invertebrates can typically be divided into two stages. In other invertebrates studied to date, the first stage of aging, studied here, appears to be set in motion by programmed life events that occur with the onset of reproduction. The second phase involves a slowly building loss of function that is associated with chronological age and the process of senescence^2–4^. In this study, we used attendants worker bees that were selected from the same frame on which the queen was found in the brood area, thus attempting to enrich for bees with a median age of ~ 8 days^19^. However, it is important to note that we did not verify worker age, which may in fact be older or younger than the target range. As worker age might contribute to the ability to mount a robust HSR, using workers of indefinite age as the point of comparison for assessing queen HSR function does introduce some uncertainty into our ability to draw conclusions. Previous findings have found conflicting results on the age-dependent changes in the steady-state expression of chaperone proteins in honey bees. Severson et al. found that the expression of Hsp70 family chaperones increases with age in worker bees^30^, while another group did not observe such changes for Hsp70 or other chaperone proteins examined^43^. Neither group attempted to look at changes in HSR potential as a function of age. Our previous work has shown that bees from the landing board of a colony (selecting for guard or forager bees that are greater than 2 weeks old^19^) still possess a robust HSR suggesting that worker maintain HSR into later stages of their life. Despite this issue, we believe the data shown here supports our model that there is no comprehensive loss of HSR function in the reproductive queens relative to workers despite this uncertainty. However, future studies will be required to more thoroughly elucidate the effect of chronological aging on worker, as well as on queen, HSR. For workers, aging can be viewed through a strict chronological lens or also in terms of life transitions. During the summer season, young worker bees perform in-hive tasks as nurse bees before a major life transition to foraging as part of the age-based structure of honey bee society known as age polyethism^19^. In ongoing studies, we are currently exploring how these life transitions impact the HSR in workers. Worker bees that emerge in the fall become winter or ‘diutinus’ bees with extended lifespans (> 4 months) that physiologically resembles nurse bees^59^. It will also be interesting to examine how any age-related changes in HSR function in workers is impacted by whether workers are summer or winter bees. Due to their long life, examination of chronological aging on the HSR in queen bees will be challenging.

The HSR is a cellular stress response which senses protein stress in the form of misfolded proteins in the cytoplasm, such as that caused by heat-shock. At the core of the response, is the transcription factor HSF, which is usually bound to the cytoplasmic chaperones HSP90 and HSC70-4, maintaining it in a monomeric, inactive form. Upon increase of unfolded proteins in the cytoplasm, HSF is activated by loss of HSP90 binding (as this molecule is sequestered by unfolded proteins). This leads to HSF trimerization, nuclear localization, and activation of genes involved in the HSR, including chaperones. In worms, age-dependent repression of the HSR occurs due to an increase in H3K27me3 marks at target gene loci, resulting in a repressed chromatin state that interferes with HSF binding and represses transcription initiation in response to HSR induction^2^. Currently, transcriptional regulation in the honey bee is incompletely understood and critical techniques to explore these factors, such as Chromatin immunoprecipitation (ChIP), DNase Hypersensitivity, and EMSA have been carried out a handful of times or not at all in the honey bee^60,61^. Further development of these tools will be critical for investigation of the molecular differences in honey bee gene regulation that allows maintenance of HSR despite the advent of reproductive potential.

Whether honey bee queens have the capacity to mount robust stress responses has important potential consequences for honey bee health. Honey bees, which provide pollination services of critical importance to humans in both agricultural and ecological settings^62^, have suffered from increased mortality at the colony level in recent years that is likely due to a complex set of interacting stresses^63^. The health and productivity of the queen is of utmost importance to overall colony function, and evidence suggests that the rates of queen failure and subsequent queen replacement are historically high^64,65^. However, the causes of queen failure are incompletely understood and likely of diverse origin. One contributing factor may be the exposure of purchased queens to stress, such as temperature extremes during shipment. In a recent study, it was found that exposure to high or low temperature extremes could result in low sperm viability^66^, which is a predictor of queen failure^67^. Our understanding of the stress tolerance and stress responses of queen bees is incomplete. The authors found a significant reduction in sperm viability after only one or 2 h at either high or low temperatures^66^. A new study suggests that the ovary tissue and the spermatheca, may possess different responses to thermal stress in the form of the types of proteins upregulated by stress^68^. There have not yet been studies examining the effect of stress on reproductive fitness independent of sperm viability, such as changes in behavior or energetics. Stress responses in non-reproductive organs, such as the brain, midgut, or fatbody, might be expected to play a key role in such changes. Our results suggest these tissues possess robust HSR function.

Here, we demonstrate that reproductive potential does not cause loss of Heat Shock Response performance in honey bees. Specifically, honey bee queens induce HSR target gene expression to levels comparable to those induced in attendant worker bees. The observed retention of HSR function with advent of reproductive potential is distinct from invertebrates studied to date and may offer a model for examining the molecular mechanisms regulating the uncoupling of the reproduction-maintenance trade-off in social insects. Our results may also have important consequences for understanding how stresses impact different castes within honey bee colonies.

## Materials and methods

### Honey bee procurement, treatment, and dissection

Newly mated honey bee queens were purchased from Betterbee (Greenwich, NY) and shipped overnight in standard queen cages (along with sterile worker bee attendants). Queens were from queen cells that were transferred to mating nucs and confirmed to be laying worker eggs approximately 21 days after addition to the nucs, making them 2–3 weeks post emergence. Attendants were selected from the same frame on which the queen was found in the brood area (as per the caging protocol at Betterbee, personal communication), enriching for bees with a median age of ~ 8 days^19^. Upon arrival at Barnard College, queen cages were given a few drops of water and allowed to acclimate to 35 °C overnight. For heat shock, caged bees were maintained for 4 h at either 35° or 45 °C. After cold anesthesia, bees were dissected, and we recovered a number of tissues for analysis: head tissue (predominantly brain and sensory organ tissue including antennae), thorax tissue (predominantly flight muscle), abdominal wall (predominantly fat body), midgut and ovarian tissue (for queens only). Tissues were stored in RNAlater (Invitrogen, San Diego, CA) at − 20 °C until extraction and further analysis. Over three independent experiments, we examined the HSR in reproductive queens and attendant sterile worker bees and combined the data for analysis and presentation. The full sample size for the combined experiments was 13 control queens /13 HS queens and 13 control attendants/13 HS attendants. Robust induction of HSR in both queens and attendants was observed in all three experiments and there was no difference in the expression levels of *Hsc70-4, Hsp70Ab, Hsp70Cb,* and *Hsp90* for queens and sterile attendant workers in the uninduced (35°) and induced (45°) states the various tissues examined for any of the independent experiments (data not shown).

### RNA isolation, reverse-transcription and quantitative PCR for gene expression analysis

RNA extraction and expression analysis was performed as previously^35^. RNA was recovered from bees by manually crushing the tissue of interest with a _disposable_ pestle in Trizol Reagent (Invitrogen, San Diego, CA) and extracting the RNA as per the manufacturer’s instructions. RNA was subsequently DNAse treated by RQ1 RNase-Free DNase (Promega, Madison, WI) and quantified. cDNA was synthesized using approximately 1 μg of RNA with the iScript cDNA Synthesis Kit (Biorad, Hercules, CA). Typically, 1 μl of cDNA was then used as a template for quantitative PCR to determine the levels of expression of genes of interest using the iQ SYBR Green Supermix (Biorad, Hercules, CA) in an iCycler thermo-cycler (Biorad, Hercules, CA). Primer sequences used in this study are in Suppl Table 1. The difference between the threshold cycle number for *β-actin* and that of the gene of interest was used to calculate the level of that gene relative to *β-actin* using the ΔΔC_T_ method.

### Statistical analysis

All gene expression data were generated by processing and analyzing individual bees (where n denotes the number of bees in each treatment group, see Statistical Data Table, Suppl Table 2.) and then pooling data for a given experiment. Graphs and Statistical Data show combined results from three independent experiments. However, analysis showed no difference in the expression levels of the examined genes for queens versus sterile attendant workers in the uninduced (35°) and induced (45°) states for the various tissues examined for any of the independent experiments (data not shown). For analysis, data was log10 transformed and compared using unpaired *t* tests with Welch’s correction as values fit normal distributions. Normality was assessed using Kolmogorov–Smirnov tests. When more than two groups were being compared, data was compared using one-way ANOVA with Tukey’s multiple comparison test. For detailed ANOVA results, see in Statistical Data Table, Suppl Table 2.

## Supplementary information

Supplementary Information.
