# Supplementary material for: Reproductive potential does not cause loss of heat shock response performance in honey bees
Source: Sci Rep. 2020 Nov 12;10:19610. doi: 10.1038/s41598-020-74456-4 (PMC7661715; doi:10.1038/s41598-020-74456-4)
Supplement: Supplementary file 1 — Supplementary Information. [file 41598_2020_74456_MOESM1_ESM.pdf]

Supplemental Materials

Reproductive potential does not cause loss of Heat Shock Response performance in honey bees

Shih, S.R. [1], Huntsman, E.M.[1], Flores, M. E. [1], and Snow J.W..[1]

[1]Biology Department, Barnard College, New York, NY, 10027, USA

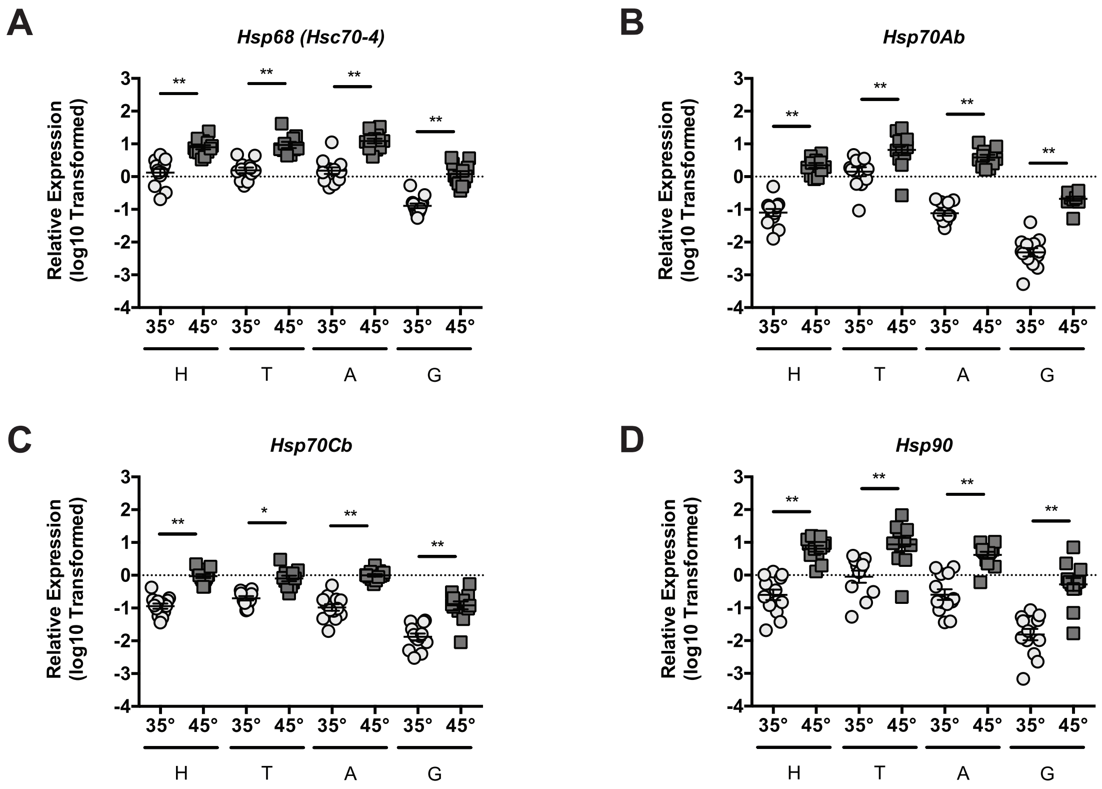

Supplemental Figure 1. HSR target genes are induced during heat-shock in sterile attendant worker bees. Transcript levels of HSR target genes *Hsc70-4* (A), *Hsp70Ab* (B), *Hsp70Cb* (C), and *Hsp90* (D), relative to *β-actin* in head tissue (H, predominantly brain and sensory organ tissue) thorax tissue (T, predominantly flight muscle), abdominal wall tissue (A, predominantly fatbody), and midgut (G) from sterile attendant worker bees maintained for four hours in cages at either 35 ° or 45 °C. Symbols represent expression values of the genes of interest calculated using the $\Delta\Delta C_T$ method for individual bees and Log10 Transformed. Individual values and mean ± SEM are also shown. Statistical significance was assessed using unpaired t-tests with Welch's correction as values fit normal distributions and is noted as $^*p < 0.05$, and $^{**}p < 0.01$.

Supplemental Table 1. Primer sequences used in this study

Name	A. mellifera Gene ID	5' primer	3' primer	Reference
**Heat shock protein cognate 4 (Hsc70-4, Hsp68)**	409418	CGATGCTGCCAAGAACCAAG	CAGCCTGAACAGTGGGATCT	this study
**Heat shock protein 70Ab-like (Hsp70Ab-like)**	410620	CTTGCGTGGGTGTTTTCCAG	TATCATCGAAGCGACGACCG	Johnston, 2016
**Heat shock protein 70Cb ortholog (Hsp110)**	408706	CATGGCTGCTATGTCCGTGA	TTTTTGGCTGCCACACCAAG	McKinstry, 2017
**Heat shock protein 90 (Hsp90)**	408928	CCATGGCTAATGCCGGAGAG	TCCGTGAGAGATTCATAGCGA	this study
**β-actin (Actin-5C, Act5C)**	406122	TTGTATGCCAACACTGTCCTTT	TGGCGCGATGATCTTAATTT	Vanengelsdorp, 2009

Supplemental Table 2. ANOVA results in Statistical Data Table

Hsc70-4 (Hsp68)

<u>Head</u>

ANOVA

Summary: F (3, 48) = 23.79, P < 0.0001

Tukey's Multiple Comparisons

	Q 35°	Q 45°	W 35°	W 45°
Q 35° (n=13)	-	**< 0.0001**	> 0.9999	**< 0.0001**
Q 45° (n=13)	**< 0.0001**	-	**< 0.0001**	> 0.9999
W 35° (n=13)	> 0.9999	**< 0.0001**	-	**< 0.0001**
W 45° (n=13)	**< 0.0001**	> 0.9999	**< 0.0001**	-

<u>Thorax</u>

ANOVA

Summary: F (3, 43) = 23.17, P < 0.0001

Tukey's Multiple Comparisons

	Q 35°	Q 45°	W 35°	W 45°
Q 35° (n=13)	-	**< 0.0001**	0.3230	**< 0.0001**
Q 45° (n=12)	**< 0.0001**	-	**0.0004**	0.7242
W 35° (n=12)	0.3230	**0.0004**	-	**< 0.0001**
W 45° (n=10)	**< 0.0001**	0.7242	**< 0.0001**	-

<u>Abdomen</u>

ANOVA

Summary: F (3, 47) = 17.42, P < 0.0001

Tukey's Multiple Comparisons

	Q 35°	Q 45°	W 35°	W 45°
Q 35° (n=12)	-	**0.0002**	0.8394	**< 0.0001**
Q 45° (n=13)	**0.0002**	-	**0.0019**	0.4529
W 35° (n=13)	0.8394	**0.0019**	-	**< 0.0001**
W 45° (n=13)	**< 0.0001**	0.4529	**< 0.0001**	-

Gut

ANOVA

Summary: F (3, 48) = 19.39, P < 0.0001

Tukey's Multiple Comparisons

	Q 35°	Q 45°	W 35°	W 45°
Q 35° (n=13)	-	**< 0.0001**	0.7722	**0.0002**
Q 45° (n=13)	**< 0.0001**	-	**< 0.0001**	0.9513
W 35° (n=13)	0.7722	**< 0.0001**	-	**< 0.0001**
W 45° (n=13)	**0.0002**	0.9513	**< 0.0001**	-

Hsp70Ab

Head

ANOVA

Summary: F (3, 48) = 66.89, P < 0.0001

Tukey's Multiple Comparisons

	Q 35°	Q 45°	W 35°	W 45°
Q 35° (n=13)	-	**< 0.0001**	0.8468	**< 0.0001**
Q 45° (n=13)	**< 0.0001**	-	**< 0.0001**	0.6559
W 35° (n=13)	0.8468	**< 0.0001**	-	**< 0.0001**
W 45° (n=13)	**< 0.0001**	0.6559	**< 0.0001**	-

Thorax

ANOVA

Summary: F (3, 45) = 13.43, P < 0.0001

Tukey's Multiple Comparisons

	Q 35°	Q 45°	W 35°	W 45°
Q 35° (n=12)	-	**< 0.0001**	0.5415	**< 0.0001**
Q 45° (n=13)	**< 0.0001**	-	**0.0028**	> 0.9999
W 35° (n=12)	0.5415	**0.0028**	-	**0.0034**
W 45° (n=12)	**< 0.0001**	> 0.9999	**0.0034**	-

Abdomen

ANOVA

Summary: F (3, 45) = 52.36, P < 0.0001

Tukey's Multiple Comparisons

	Q 35°	Q 45°	W 35°	W 45°
Q 35° (n=11)	-	**< 0.0001**	0.9474	**< 0.0001**
Q 45° (n=13)	**< 0.0001**	-	**< 0.0001**	0.1330
W 35° (n=13)	0.9474	**< 0.0001**	-	**< 0.0001**
W 45° (n=12)	**< 0.0001**	0.1330	**< 0.0001**	-

Gut

ANOVA

Summary: $F(3, 47) = 85.38$, $P < 0.0001$

Tukey's Multiple Comparisons

	Q 35°	Q 45°	W 35°	W 45°
Q 35° (n=13)	-	**< 0.0001**	0.9344	**< 0.0001**
Q 45° (n=13)	**< 0.0001**	-	**< 0.0001**	0.8423
W 35° (n=13)	0.9344	**< 0.0001**	-	**< 0.0001**
W 45° (n=12)	**< 0.0001**	0.8423	**< 0.0001**	-

Hsp70Cb

Head

ANOVA

Summary: $F (3, 48) = 44.44$, $P < 0.0001$

Tukey's Multiple Comparisons

	Q 35°	Q 45°	W 35°	W 45°
Q 35° (n=13)	-	**< 0.0001**	0.5006	**< 0.0001**
Q 45° (n=13)	**< 0.0001**	-	**< 0.0001**	0.9277
W 35° (n=13)	0.5006	**< 0.0001**	-	**< 0.0001**
W 45° (n=13)	**< 0.0001**	0.9277	**< 0.0001**	-

Thorax

ANOVA

Summary: $F (3, 43) = 16.21$, $P < 0.0001$

Tukey's Multiple Comparisons

	Q 35°	Q 45°	W 35°	W 45°
Q 35° (n=12)	-	**< 0.0001**	0.9566	**< 0.0001**
Q 45° (n=12)	**< 0.0001**	-	**0.0001**	> 0.9999
W 35° (n=12)	0.9566	**0.0001**	-	**0.0002**
W 45° (n=11)	**0.0002**	> 0.9999	**< 0.0001**	-

Abdomen

ANOVA

Summary: $F (3, 45) = 24.94$, $P < 0.0001$

Tukey's Multiple Comparisons

	Q 35°	Q 45°	W 35°	W 45°
Q 35° (n=11)	-	**< 0.0001**	0.9249	**< 0.0001**
Q 45° (n=12)	**< 0.0001**	-	**< 0.0001**	> 0.9999
W 35° (n=13)	0.9249	**< 0.0001**	-	**< 0.0001**
W 45° (n=13)	**< 0.0001**	> 0.9999	**< 0.0001**	-

<u>**Gut**</u>

ANOVA

Summary: $F(3, 52) = 35.61$, $P < 0.0001$

Tukey's Multiple Comparisons

	Q 35°	Q 45°	W 35°	W 45°
Q 35° (n=13)	-	**< 0.0001**	0.8880	**< 0.0001**
Q 45° (n=13)	**< 0.0001**	-	**< 0.0001**	0.2015
W 35° (n=13)	0.8880	**< 0.0001**	-	**< 0.0001**
W 45° (n=13)	**< 0.0001**	0.2015	**< 0.0001**	-

Hsp90

Head

ANOVA

Summary: $F (3, 48) = 30.55$, $P < 0.0001$

Tukey's Multiple Comparisons

	Q 35°	Q 45°	W 35°	W 45°
Q 35° (n=13)	-	**< 0.0001**	0.7965	**< 0.0001**
Q 45° (n=13)	**< 0.0001**	-	**< 0.0001**	0.9954
W 35° (n=13)	0.7965	**< 0.0001**	-	**< 0.0001**
W 45° (n=13)	**< 0.0001**	0.9954	**< 0.0001**	-

Thorax

ANOVA

Summary: $F (3, 42) = 10.51$, $P < 0.0001$

Tukey's Multiple Comparisons

	Q 35°	Q 45°	W 35°	W 45°
Q 35° (n=12)	-	**0.0010**	0.9228	**0.0004**
Q 45° (n=12)	**0.0010**	-	**0.0077**	0.9751
W 35° (n=11)	0.9228	**0.0077**	-	**0.0030**
W 45° (n=11)	**0.0004**	0.9751	**0.0030**	-

Abdomen

ANOVA

Summary: $F (3, 44) = 18.21$, $P < 0.0001$

Tukey's Multiple Comparisons

	Q 35°	Q 45°	W 35°	W 45°
Q 35° (n=11)	-	**0.0003**	0.6792	**0.0004**
Q 45° (n=11)	**0.0003**	-	**< 0.0001**	0.9940
W 35° (n=13)	0.6792	**< 0.0001**	-	**< 0.0001**
W 45° (n=13)	**0.0004**	0.9940	**< 0.0001**	-

<u>**Gut**</u>

ANOVA

Summary: F (3, 51) = 25.22, P < 0.0001

Tukey's Multiple Comparisons

	Q 35°	Q 45°	W 35°	W 45°
Q 35° (n=13)	-	**< 0.0001**	0.5215	**< 0.0001**
Q 45° (n=13)	**< 0.0001**	-	**< 0.0001**	0.9591
W 35° (n=13)	0.5215	**< 0.0001**	-	**< 0.0001**
W 45° (n=12)	**< 0.0001**	0.9591	**< 0.0001**	-
